# DHOK Exerts Anti-Cancer Effect Through Autophagy Inhibition in Colorectal Cancer

**DOI:** 10.3389/fcell.2021.760022

**Published:** 2021-12-17

**Authors:** Yuhan Shu, Xin Sun, Guiqin Ye, Mengting Xu, Zhipan Wu, Caixia Wu, Shouxin Li, Jingkui Tian, Haote Han, Jianbin Zhang

**Affiliations:** ^1^ College of Biomedical Engineering and Instrument Science, Zhejiang University, Hangzhou, China; ^2^ Department of Oncology, Cancer Center, Key Laboratory of Tumor Molecular Diagnosis and Individualized Medicine of Zhejiang Province, Zhejiang Provincial People’s Hospital, People’s Hospital of Hangzhou Medical College, Hangzhou, China; ^3^ Hangzhou Medical College, Hangzhou, China; ^4^ The Cancer Hospital of the University of Chinese Academy of Sciences (Zhejiang Cancer Hospital), Institute of Basic Medicine and Cancer (IBMC), Chinese Academy of Sciences, Hangzhou, China

**Keywords:** DHOK, transcriptome sequencing, autophagy, colorectal cancer, mTOR pathway

## Abstract

DHOK (14,15β-dihydroxyklaineanone) is a novel diterpene isolated from roots of *Eurycoma longifolia Jack*, a traditional herb widely applied in Southeast Asia. It is reported that DHOK has cytotoxic effect on cancer cells, but its anti-cancer mechanism has still been not clear. In our study, we first observed that DHOK inhibits cell proliferation of colorectal cancer cells in a time- and dose-dependent manner. Next, we performed transcriptome sequencing to identify the targets of DHOK and found that autophagy-related signaling pathways are involved under DHOK treatment. Indeed, in DHOK-treated cells, the level of autophagosome marker LC3 and the formation of GFP-LC3 puncta were decreased, indicating the reduction of autophagy. Moreover, confocal microscopy results revealed the lysosomal activity and the formation of autolysosomes are also inhibited. Our western blotting results demonstrated the activation of mammalian target of rapamycin (mTOR) signaling pathway by DHOK, which may be attributed to the enhancement of ERK and AKT activity. Functionally, activation of autophagy attenuated DHOK-caused cell death, indicating that autophagy serves as cell survival. In xenograft mouse model, our results also showed that DHOK activates the mTOR signaling pathway, decreases autophagy level and inhibits the tumorigenesis of colon cancer. Taken together, we revealed the molecular mechanism of DHOK against cancer and our results also demonstrate great potential of DHOK in the treatment of colorectal cancer.

## Introduction

Colorectal cancer (CRC), a common malignant tumor of the digestive tract, mainly occurs in the colon or rectum. Among all cancers, CRC is the third most commonly diagnosed cancer (10.2% of total cases), and the third case of death (9.2% of total cancer deaths) (Bray et al., 2018). By sex, CRC is the third most commonly diagnosed cancer (10.9%) and the fourth most cause of cancer death (9.0%) in males. Among females, CRC cancer is the second most commonly diagnosed cancer (9.5%) and the third cause of cancer death (9.5%). Despite the general diminishments of CRC incidence for adults age >50 because of schedule screening, CRC incidence among adults <50 proceeds on expansion (Venugopal and Stoffel, 2019). Following by complete surgical resection, standard chemotherapy and targeted therapy are the most common treatments to CRC (Cespedes Feliciano et al., 2016). Current therapies to CRC mainly depend on customary cytotoxic agents, but they not only have limited effect to CRC but also cause damage effect to human health (Lee et al., 2015).

**FIGURE 1:**
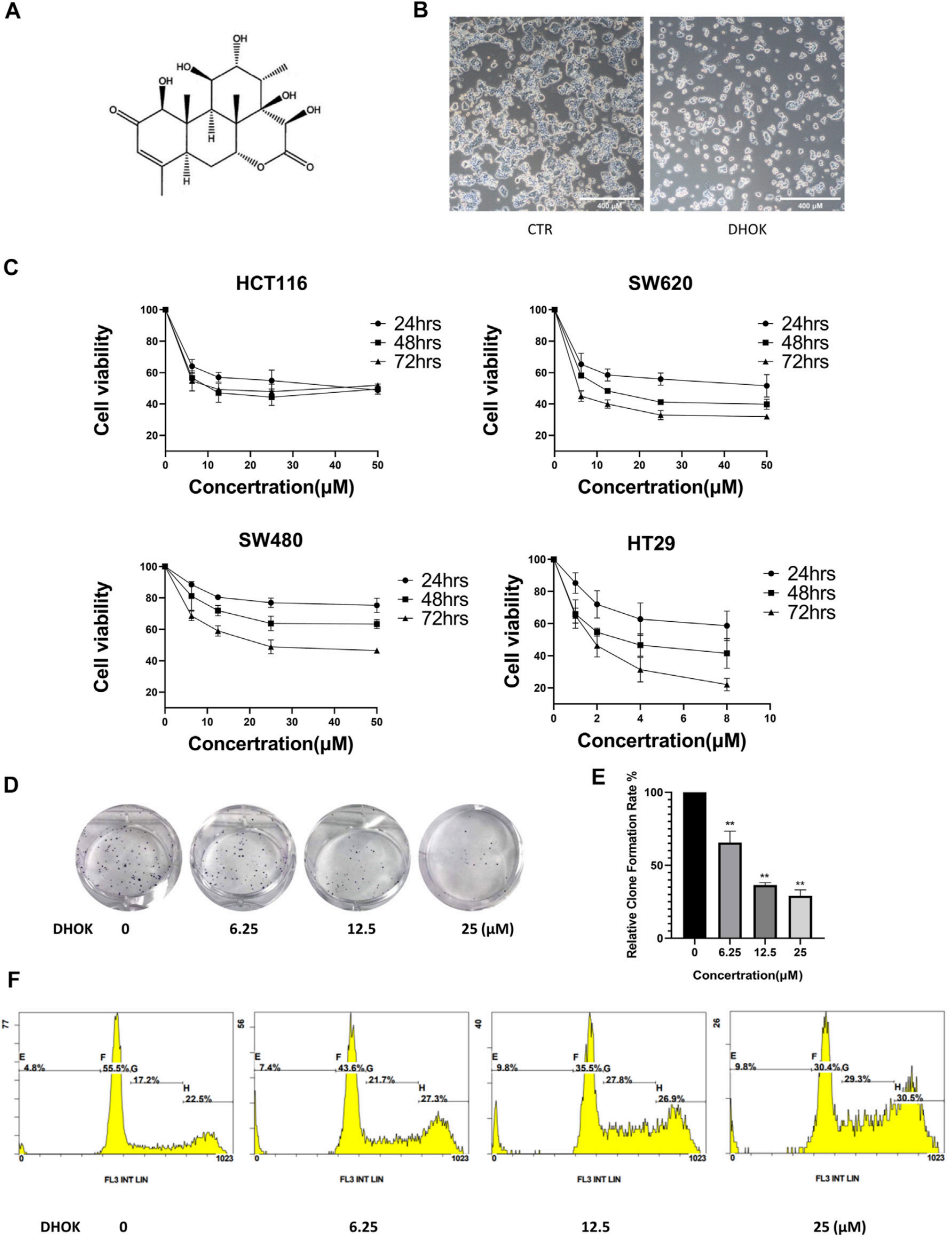
DHOK inhibits human CRC cell proliferation. **(A)** The structure of DHOK. **(B)** Cell morphology of SW620 cells with or without DHOK treatment (12.5 μM, 24 h). Scale bar 400 μm. **(C)** Cell viability of SW620, SW480, HCT116, and HT29 cells under different dosage of DHOK treatment (6.25, 12.5, 25 μM; or 1, 2, 4, 8 μM) for 24, 48, or 72 h **(D,E)** Colony formation assay of SW620 cells after different dosage of DHOK treatment (6.25, 12.5, 25 μM). The number of formed colonies was counted and statistically analyzed **p < 0.01. **(F)** The cell cycle of SW620 cells was analysed under DHOK treatment.

Eurycoma longifolia *Jack* (known as tongkat ali) is commonly found along the hilly jungle slopes in Southeast Asia, including Malaysia, Burma, Indochina, Thailand, Indonesia, and Philippines. *E. longifolia Jack* is a traditional herb used as traditional treatments for dysentery, fever, persistent fever, malaria, syphilis, smallpox, washing itches, and sexual insufficiency (Mohd-Fuat et al., 2007). Studies have proved that the extract of *E. longifolia Jack* has effects such as anti-tumor (Lau et al., 2009), anti-inflammatory (Ruan et al., 2019), anti-HIV (Kuo et al., 2004), and anti-gout (Liu et al., 2019). Eurycoma longifolia has various classes of bioactive compounds including quassinoids, β-carboline alkaloids, canthin-6-onealkaloids, triterpene-typetirucallane, squalenederivatives, andeurycolactone, eurycomalactone, laurycolactone, biphenyl neolignan, and bioactive steroids (Ang et al., 2002; Kuo et al., 2004; Bhat and Karim, 2010). DHOK (14,15β-dihydroxyklaineanone) (Structure is shown in Figure 1A) is a diterpene isolated from *E. longifolia Jack*, which has cytotoxicity to cancer cells, including human lung cancer, and human breast cancer (Kuo et al., 2004). However, the detailed anticancer mechanisms of DHOK remain unknown.

Autophagy is an evolutionarily conserved transport pathway which sequestrates, unwanted proteins, macromolecular complexes, and organelles into lysosomes for degrdation to maintain cellular homeostasis (Mauthe et al., 2018). At the early stage of autophagy, it is characterized with the formation of autophagosomes, which are double-membrane vesicles engulfing cytoplasmic constituents; at the late stage of autophagy, autophagosomes fuse with lysosomes to form autolysosomes to degrade the sequestered contents (Yu et al., 2018). Autophagy plays a dual role and has an association with apparently contradictory roles, such as pro-survival and pro-death (Dalby et al., 2010). Among plenty of pathways, mTOR pathway is one of the key pathways that regulate autophagy. High mTOR activity prevents Unc-51 Like Autophagy Activating Kinase 1 (ULK1) activation by phosphorylating ULK1 at Ser 757, and then inhibits autophagy initiation (Kim et al., 2011).

MTOR, a serine/threonine protein kinase, belongs to the phosphatidylinositol 3-kinases (PI3K) family. The activity of mTOR is inhibited under nutrient starvation, which is a crucial step of autophagy induction in eukaryotes (Jung et al., 2010). mTOR is also a central cell-growth regulator, which integrates growth factors and nutrient signals and inhibits autophagy (Kim et al., 2011). It is believed that the activated mTOR supports certain tumor cell growth by promoting protein synthesis, which is also the most important function of mTOR signaling (Agostini et al., 2018). The upstreams of mTOR signaling include PI3K-protein kinase B (AKT) pathway, the adenosine monophosphate-activated protein kinase (AMPK) pathway and the rat sarcoma (Ras)/rapidly accelerated fibrosarcoma (Raf)/mitogen-extracellular activated protein kinase (MEK)/ extracellular-signal-regulated kinase (ERK) pathway (Wang et al., 2019). AMPK activates autophagy though activating the ULK1 by phosphorylating Ser 317 and Ser 777 under glucose starvation. Howerver, high mTOR activity prevents ULK1 activation by phosphorylating ULK1 at Ser 757, and then inhibits autophagy initiation (Kim et al., 2011). AKT and ERK active mTOR signaling by inhibiting tuberous sclerosis complex (TSC)-1 and -2, which negatively regulates mTOR activity (Huang et al., 2008).

In this study, we detailedly explore the anti-cancer mechanism of DHOK in human CRC. Our results demonstrate that DHOK inhibits human CRC cell growth and the tumorigenesis of human CRC. It may be attributed to cell cycle arrest and the suppression of autophagy by DHOK. It was also revealed that DHOK treatment activates mTOR signaling pathway to decrease autophagy level both *in vitro* and *in vivo*. Moreover, the function of the autophagy inhibited by DHOK is pro-survival. These findings indicate that DHOK might be developed to be a novel therapeutic agent for human CRC though inhibiting protective autophagy.

## Materials and Methods

### Reagents and Antibodies

These following reagents were used in our research: crystal violet (Beyotime, #C0121), MTT (Solarbio, #M8180), dimethyl sulfoxide (DMSO, Sangon biotech, #A503039), a BCA protein assay kit (Solarbio, #PC0020), chloroquine (CQ, Sigma-Aldrich, #C6628), LysoTracker Red DND-99 (Invitrogen, #L7528), fetal bovine serum (FBS, Biological Industries, #04-001-1A), trypsin-EDTA Solution (BBI, #E607001), Earle’s balanced salts solution (EBSS, Gibco, #24010043), BeyoECL Plus (Beyotime, #P0018S), and Lipofectamine 2000 reagent (Invitrogen, #11668027).

Antibodies were obtained as follows: anti-LC3 antibody (Sigma-Aldrich, L7543), anti-α-tubulin (Sigma-Aldrich, T6199), anti-FLAG (Sigma-Aldrich, F3165), anti-β-actin (Sigma-Aldrich, A5441), anti-phospho-JNK (Proteintech, 80024) and anti-JNK (Proteintech, 66210). All of the other antibodies were purchased from Cell Signaling Technology: anti-phospho-AKT (cata. no. 4060), anti-AKT (cata. no. 4691), phospho-mTOR (cata. no.5536), anti-mTOR (cata. no. 2983), anti-phospho-S6 (cata.no. 2211), anti-S6 (cata. no. 2217), anti-phospho-4E-BP1 (cata.no. 2855), anti-4E-BP1(cata.no. 9452), anti-ERK1/2 (cata.no. 9102), anti-phospho-ERK1/2 (cata.no. 9101), anti-TSC2 (cata.no. 4308), anti-phospho-p38 (cata.no. 4511) and anti-p38 (cata.no 8690).

### Cell Culture

SW620 cells, SW480 cells, HCT116, and HT29 cells were purchased from ATCC (American Type Culture Collection). HeLa cells stably expressing GFP-LC3 and L929 cells stably expressing tfLC3 were kindly provided by Professor Shen Han-Ming (National University of Singapore, Singapore). Cells were maintained in high glucose dulbecco’s modified eagle medium (DMEM, HyClone) and supplemented with 10% FBS, 100 units/ml penicillin, 100 mg/ml streptomycin, 1 mM sodium pyruvate, and 1 mM nonessential amino acids. Cells were grown in 75-cm^2^ cell culture flasks (Biofil) at 37°C in a 5% CO_2_ incubator (Thermo Scientific).

### Cell Viability Assay

Cell viability was measured using the MTT assay. Cells (3 × 10^3^) were seeded in 96-well plates and allowed to attach overnight in a 5% CO_2_ incubator. The cells were then treated with DHOK (6.25, 12.5, 25, 50, and 100 μM). MTT (2.5 mg/ml) was added to each well and incubated for another 2 h at 37°C. The supernatants were carefully aspirated, and the purple formazan crystals were dissolved in 200 µL of DMSO. The absorbance was measured by a Multiscan spectrophotometer (Thermo Scientific) at 570 nm.

### Colony Formation Assay

A total of five hundred cells were seeded in a 6-well plate for 5 days. The original medium was removed, and the cells were incubated in medium containing DHOK at each concentration for 48 h. Then the medium was changed and incubated for another 14 days. Colonies were washed with PBS for three times and fixed by 4% paraformaldehyde (PFA), and then was photographed after staining with 0.1% (w/v) crystal violet. ImageJ was used to count colonies.

### RNA Sequencing

SW620 cells were seeded in 6-cm dishes overnight and treated with 12.5 μM DHOK for 24 h in a 5% CO_2_ incubator. The next day, 1 ml Trizol buffer (Beyotime, Shanghai, China) was used to lyse cells following the manufacturer’s procedure (Sun et al., 2020). The RNA sequencing (RNA-seq) experiments were undertaken by Lc-Bio Technologies Co., Ltd. (Hangzhou, China). The amount and purity of RNA was quantified by NanoDrop ND-1000 (NanoDrop, Wilmington, DE, United States). Then RNA was purified and fragmented. After that, RNA was reverse transcribed to cDNA, and next synthesized U-labeled second-stranded DNAs. An A-base was then added to the blunt ends of each strand and single- or dual-index adapters were ligated to the fragments. AMPureXP beads were used to size selection. The average insert size for the final cDNA library was 300 ± 50 bp. At last, the paired-end sequencing was performed.

### Transmission Electron Microscopy

SW620 cells were seeded into 6-cm dishes. After 24 h, cells were co-treated with 12.5 μM DHOK and 100 nM rapamycin (which induces autophagy though inhibiting the activity of mTOR) for 24 h. Then, the cells were collected with a cellscraper and centrifuged at 2,000 rpm for 4°C. The collected cells were fixed with 4% glutaraldehyde for another 2 h at room temperature and stored at 4°C (Gao et al., 2020). Electron photomicrographs of the SW620 cells were taken (Wuhan Goodbio Technology Co., Ltd., Wuhan, China).

### Lysosomal Morphology

HeLa-GFP-LC3 cells were seeded in eight-well Lab-Tek™ chambered coverglass (Thermo Scientific, 155411) overnight. Then, cells were co-treated with 12.5 μM DHOK and 100 nM rapamycin for 24 h. After that, supernatants were discarded and the cells were washed with PBS. Cell were stained with lysotracker red for 30 min. Confocal microscope was used to observe lysosome.

### Western Blotting

Cells were seeded into 6-well plates or 12-well plates for 24 h and treated with DHOK. 24 h later, cells were collected by scraping and washed three times with PBS. Then, the cells were lysed with RIPA buffer for 30 min on ice. The mixture was centrifuge at 13,200 rpm for 15 min at 4°C. The protein concentration in the supernatant were determined by a BCA protein assay kit (Beyotime, P0012). Equal amounts of proteins were separated on SDS-polyacrylamide gels and then electroblotted onto polyvinylidene fluoride (PVDF) membranes (Bio-Rad, 1620184). The membranes were blocked with Quick Block blocking buffer (Beyotime, P0252-500 ml) for more than 10 min and incubated with primary antibodies (1:1,000) overnight at 4°C. The next day, TBST was used to wash the membranes for 10 min × 3 times. Then, the membranes were incubated with secondary antibodies (1:3,000) for 2 h at room temperature. The membranes were washed 10 min × 3 times before development, and the immunoblots were visualized with an ECL system.

### Confocal Imaging

Cells were cultured in eight-well Lab-Tek™ chambered coverglass (Thermo Scientific, 155411) overnight and treated. All of the confocal images were obtained with × 60 oil objective (numerical aperture 1.4) lenses of Leika TCS SP5 Confocal.

### Cell Transfection

SW620 and HEK293 cells were seeded into 6-well plates overnight. Then, empty vector and Flag-Beclin1 plasmid were transfected into cells using Lipofectamine 2000 according to the manufacturer’s protocol. After 24 h, the cells were digested with trypsin, and collected by centrifuging. Then cells were reseeded for following experiments.

### Cell Cycle Analysis

SW620 cells were seeded into 6-well plates overnight. The next day, cells were treated with DHOK (0, 6.25, 12.5, and 25 μM). After 48 h treatment, cells were digested and washed. After that, cells were resuspended with 1 ml 70% ice-cold alcohol. Then cells were fixed at 4°C overnight. The next day, cells were washed with ice-cold PBS. Subsequently, cells were stained with RNase A-containing propidium iodide (PI) at 37°C for 30 min. Cell cycle phases were analyzed by flow cytometry at 488 nm.

### *In Vivo* Study

Female Balb/c nude mice (3–4 weeks old) were purchased from the Shanghai SLAC Laboratory Animal Co. Ltd. (Shanghai, China). Animal welfare was ensured, and the experimental procedures strictly followed the Guide for the Care and Use of Laboratory Animals. All efforts were made to minimize animal suffering and to reduce the number of animals used. Xenograft tumors were established by subcutaneously injecting 1 × 10^7^ SW620 cells in PBS in a total volume of 0.1 ml. After 7 days, tumor-bearing mice were randomly divided into 2 groups: the vehicle group and DHOK (40 mg/kg) treatment group. The Vehicle group was treated with 10% DMSO, 20% polyethylene glycol and 5% Tween-80 in PBS (Shu et al., 2021). Two groups received treatments every 2 days by intraperitoneal injection. Mice were sacrificed after another 2 weeks. Tumor volume was calculated by the formula: Tumor volume = Length × Width × Width × 0.5.这个公式对的?

### Statistical Analysis

All experiments were repeated for three times. The results were analyzed using one-way ANOVA for statistical significance. Data were expressed as the mean ± standard error. *p < 0.05, **p < 0.01, ***p < 0.001. As long as *p*-value < 0.05, the difference was considered statistically significant.

## Results

### DHOK Inhibits Cell Proliferation in Human CRC Cells

To evaluate the anti-proliferative activity of DHOK in human CRC cells, SW620 cells were treated with DHOK and cell morphology showed cell blebbing and shrinkage, accompanied with a significant decrease of cell number (Figure 1B). In addition, several CRC cell lines were exposed to DHOK at different concentrations for 24, 48, and 72 h (Figure 1C), including SW620, SW480, HCT116, and HT29 cells. As shown in Figure 1C, the cytotoxicity of DHOK was shown a dose- and time-dependent manner. The IC_50_ of DHOK for SW620, SW480, and HCT116 cells were 12.35 μM (48 h), 28.99 μM (72 h), and 17.52 μM (48 h), respectively, while HT29 cells were much more sensitive to DHOK and the IC_50_ was around 4 μM (48 h). Next, we determined the inhibitory effect of DHOK on cell proliferation using colony formation assay. The results showed that DHOK significantly decreased colony formation in SW620 cells (Figures 1D,E). In order to know whether apoptosis is related with the anti-cancer effect of DHOK, we examined cell apoptosis by flow cytometryand found that DHOK did not increase the apoptosis level in SW620 cells (Supplementary Figure S1A). And cell cycle analysis was performed and showed that DHOK caused G2/M phase arrest in SW620 cells (Figure 1F), which may be the reason for the cell growth-inhibitory effect of DHOK.

### Profiling of DHOK Targets Through Transcriptome Sequencing

**FIGURE 2:**
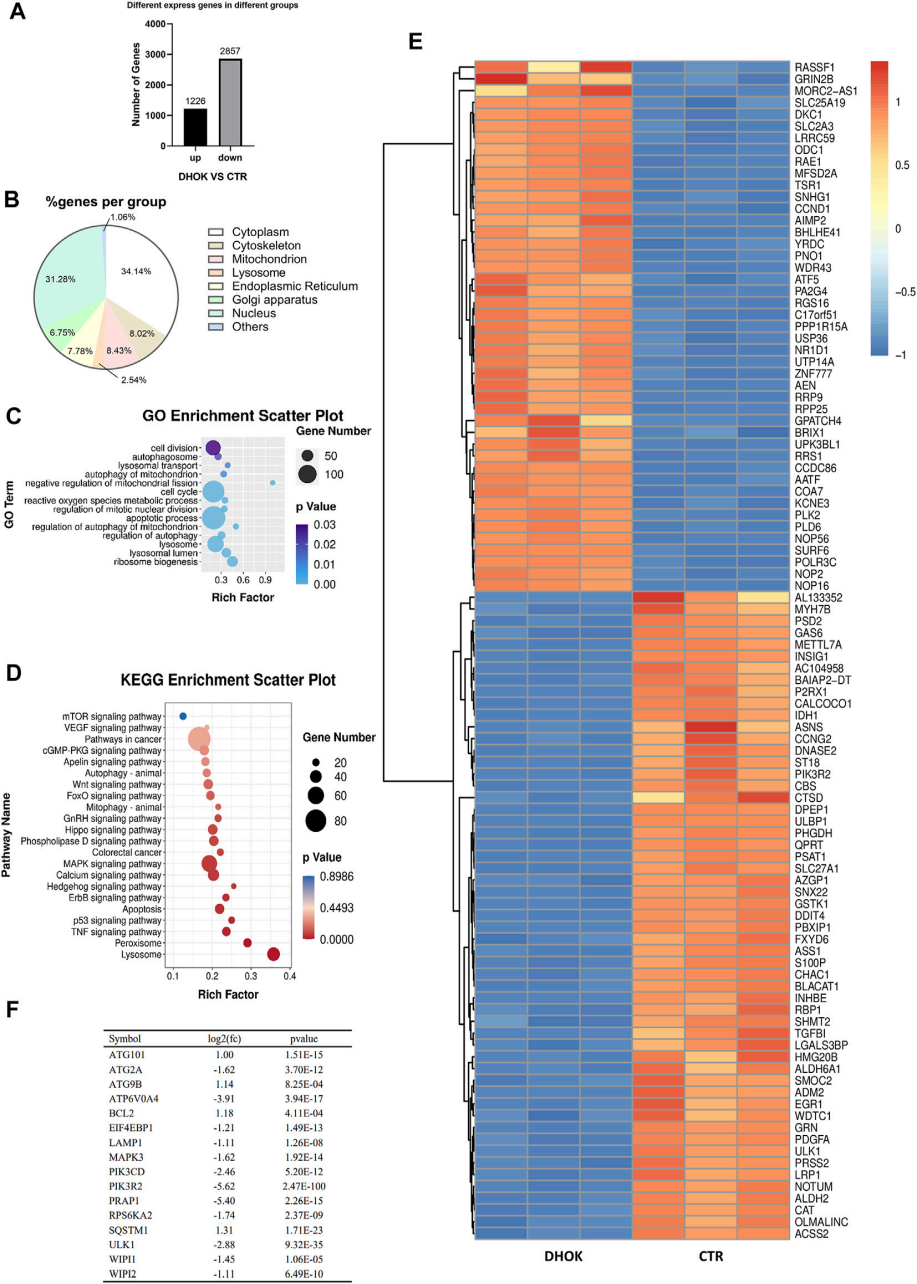
DHOK exerts an inhibitory effect on autophagy-related signaling pathway. SW620 cells were treated with DHOK (12.5 μM, 24 h) and harvested for RNA extraction. Human gene expression was analysed using transcriptome sequencing. **(A)** The number of significant differential expressed gene. **(B)** Cellular localization of the DHOK targets was shown in GO analysis. **(C)** GO enrichment analysis scatter plot. The size of black circle was meant to the unigene number and different colors were meant to different *p* values. **(D)** KEGG enrichment of signaling pathways in DHOK-treated cells. Red and blue color refered to the upregulated and downregulated genes, respectively. The fold changes were also described on the top-right corner. **(E)** Heat map of the differentially expressed genes. **(F)** List of autophagy-related genes that have significant changes.

To elucidate the anti-cancer mechanism of DHOK, we used transcriptome sequencing (RNA-seq) to identify cancer-associated gene signatures in human CRC cells treated with DHOK. The differential expression of genes in SW620 cells treated with or without DHOK (12.5 μM, 24 h) was significant. Compared with the control, there were totally 4083 genes with their expression levels significantly altered following DHOK treatment. Among these differentially expressed genes, 2857 genes were downregulated and 1226 genes were upregulated (Figure 2A). Subsequently, we performed gene ontology (GO) analysis of DHOK targets. It was shown that the distribution of these targets was in different parts of the cell, and especially in the nucleus, cytoplasm, mitochondria, lysosome, endoplasmic reticulum, Golgi apparatus etc. (Figure 2B).

To understand the inhibitory effect of DHOK on human CRC cells, the differentially expressed genes identified were subjected to functional enrichment analysis. The significantly enriched GO terms were mainly associated with autophagy, including autophagosome formation, lysosomal transport, mitophagy, regulation of autophagy, metabolic stress etc. (Figure 2C). Kyoto encyclopedia of genes and genomes (KEGG) analysis showed that the targets of DHOK participated in various signaling pathways, including vascular endothelial growth factor (VEGF), mTOR, P53, tumor necrosis factor (TNF-α), mitogen activated protein kinase (MAPK), Ras, Wnt, forkhead box (FoxO) etc. (Figure 2D).

The GO enrichment analysis also indicated that autophagy-related genes underwent significant changes in response to DHOK treatment. In the significantly altered genes, 45 genes were upregulated and 55 genes were downregulated (Figure 2E). Among those significantly changed genes, many genes belong to the core autophagy machinery and plays an essential role in autophagosome formation. As shown in Figure 2F, the autophagy level was decreased with the downregulation of series of autophagy-related genes, including WD repeat domain phosphoinositide interacting (WIPI)1, WIPI2, autophagy related 2A (ATG2A), ATPase H^+^ transporting V0 subunit A4 (ATP6V0A4) etc.

### DHOK Inhibits Autophagy in Human CRC Cells

**FIGURE 3:**
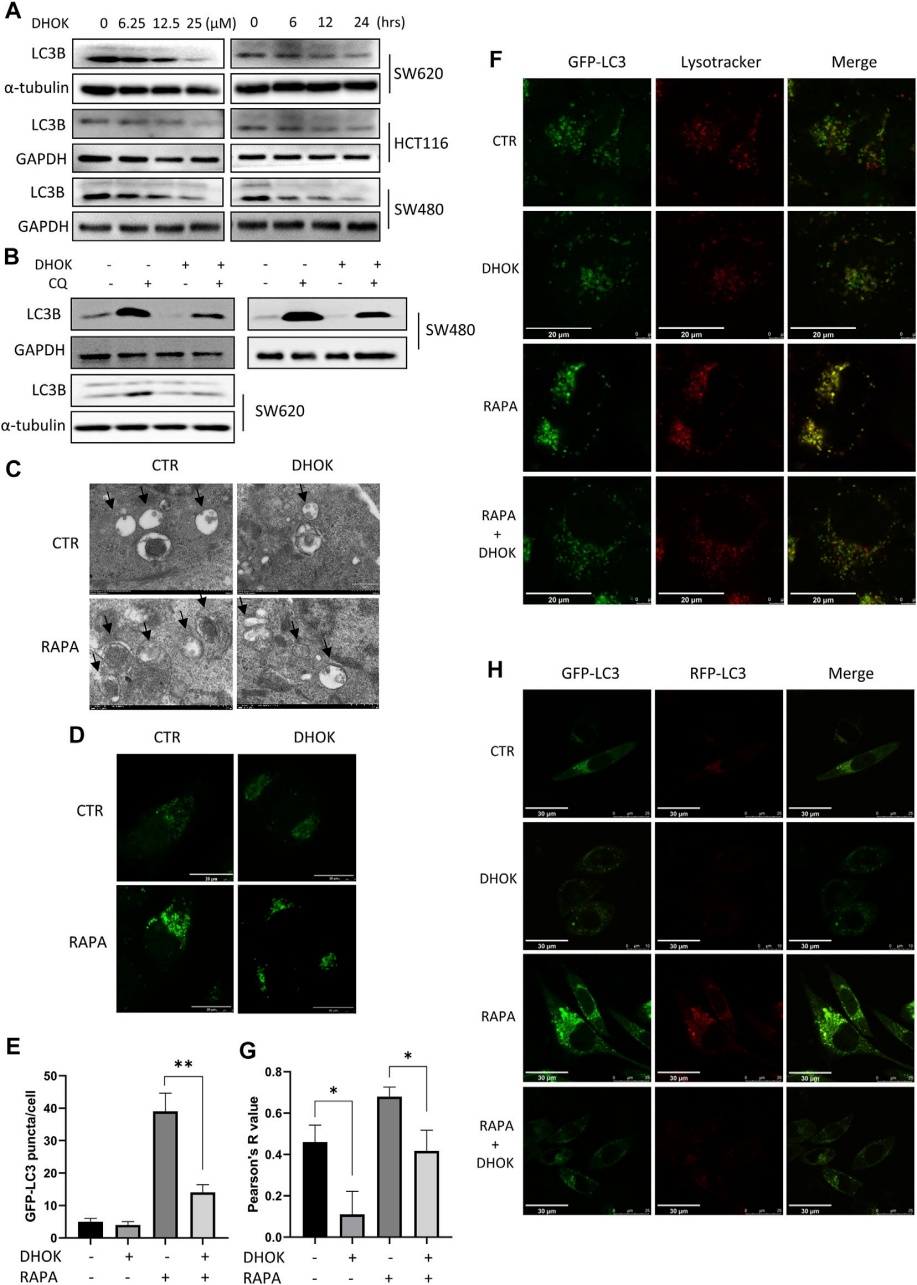
DHOK reduced autophagy level and inhibited the fusion of autophagosomes with lysosomes. **(A)** Expression of the LC3 protein in SW620, HCT116, and SW480 cells under different dosage of DHOK treatment (6.25, 12.5, 25 μM) for different time (6, 12, 24 h) was determined. **(B)** Expression of the LC3 protein in HCT116, SW480 and SW620 cells under DHOK treatment (12.5 μM, 24 h) with or without CQ (10 μM) was determined. **(C)** TEM images showed the ultrastructure of SW620 cells under DHOK treatment (12.5 μM, 24 h) in the presence or absence of rapamycin (100 nM). Scale bar 0.2 μm. **(D,E)** GFP-LC3 stably expressing cells were treated with DHOK (12.5 μM, 24 h) in the presence of rapamycin (100 nM) for 24 h. Confocal images of GFP-LC3 puncta were photographed. Scale bar 20 µm. The number of GFP-LC3 puncta were quantified by ImageJ. **p < 0.01 **(F,G)** HeLa cells stably expressing GFP-LC3 were stained with LysoTracker Red under DHOK treatment (12.5 μM, 24 h) in the presence of rapamycin (100 nM) for 24 h. Confocal images of GFP-LC3 puncta and lysosomes were photographed. Scale bar 20 µm. Colocalization index was calculated by ImageJ software. **(H)** L929 cells stably expressing tfLC3B were treated with DHOK (12.5 μM, 24 h) in the presence or absence of rapamycin (100 nM). Confocal images of GFP-LC3 and RFP-LC3 puncta were photographed (scale bar: 30 µm).

To clarify the effect of DHOK on autophagy, cells were first treated with DHOK and the level of microtubule-associated protein 1 light chain 3 (LC3, autophagosome marker) was assessed. As shown in Figure 3A, the protein levels of LC3 were decreased in DHOK-treated cells, including SW620, HCT116, and SW480 cells. Moreover, autophagic flux level was also determined and CQ was used to inhibit lysosomal activity. In the presence of autophagy inhibitor CQ, the level of LC3 in these cell lines was also decreased by DHOK (Figure 3B), indicating the attenuated autophagic flux. In addition, transmission electron microscopy was also used for the assessment of the ultrastructure of DHOK-treated SW620 cells. As shown in Figure 3C, autophagy inducer rapamycin led to a large number of double-membrane autophagosomes formation, but the addition of DHOK markedly decreased the formation of autophagosomes. Meanwhile, confocal microscopy results also showed that DHOK treatment significantly attenuated the increase of GFP-LC3 puncta formation by rapamycin (Figures 3D,E).

Next, we investigated the effect of DHOK on the lysosomal function, which belongs to the late stage of autophagic process. HeLa cells stably expressing GPF-LC3 was first treated with autophagy inducer rapamycin and then exposed to DHOK treatment. After LysoTracker Red staining, cells were imaged under a confocal microscope and we observed less lysosomes in DHOK-treated cells with red puncta, indicating the reduction of lysosomal acidification. While rapamycin treatment significantly increased the fluorescence levels of GFP-LC3 and lysosomes, and more importantly, the colocalization of GFP-LC3 and lysosomes were also enhanced. But the addition of DHOK attenuated the increase of lysosomal acidification and the fusion of lysosomes with autophagosomes by rapamycin (Figures 3F,G), indicating the inhibition of lysosomal function of DHOK. In addition, we also used L929 cells expressing mRFP-GFP tandem fluorescent-tagged LC3B (tfLC3) to examine the formation of autolysosomes. Under the acidic environment of the lysosome, the GFP-LC3 is degraded while the RFP-LC3 is stable. Thus, the number of RFP-LC3 puncta is often regarded as an indicator of autolysosome formation. As shown in Figure 3H, we observed the increase of RFP-LC3 puncta by rapamycin was also attenuated with the addition of DHOK, confirming the declined formation of autolysosomes.

### DHOK Treatment Leads to the Activation of mTOR Signaling Pathway

**FIGURE 4:**
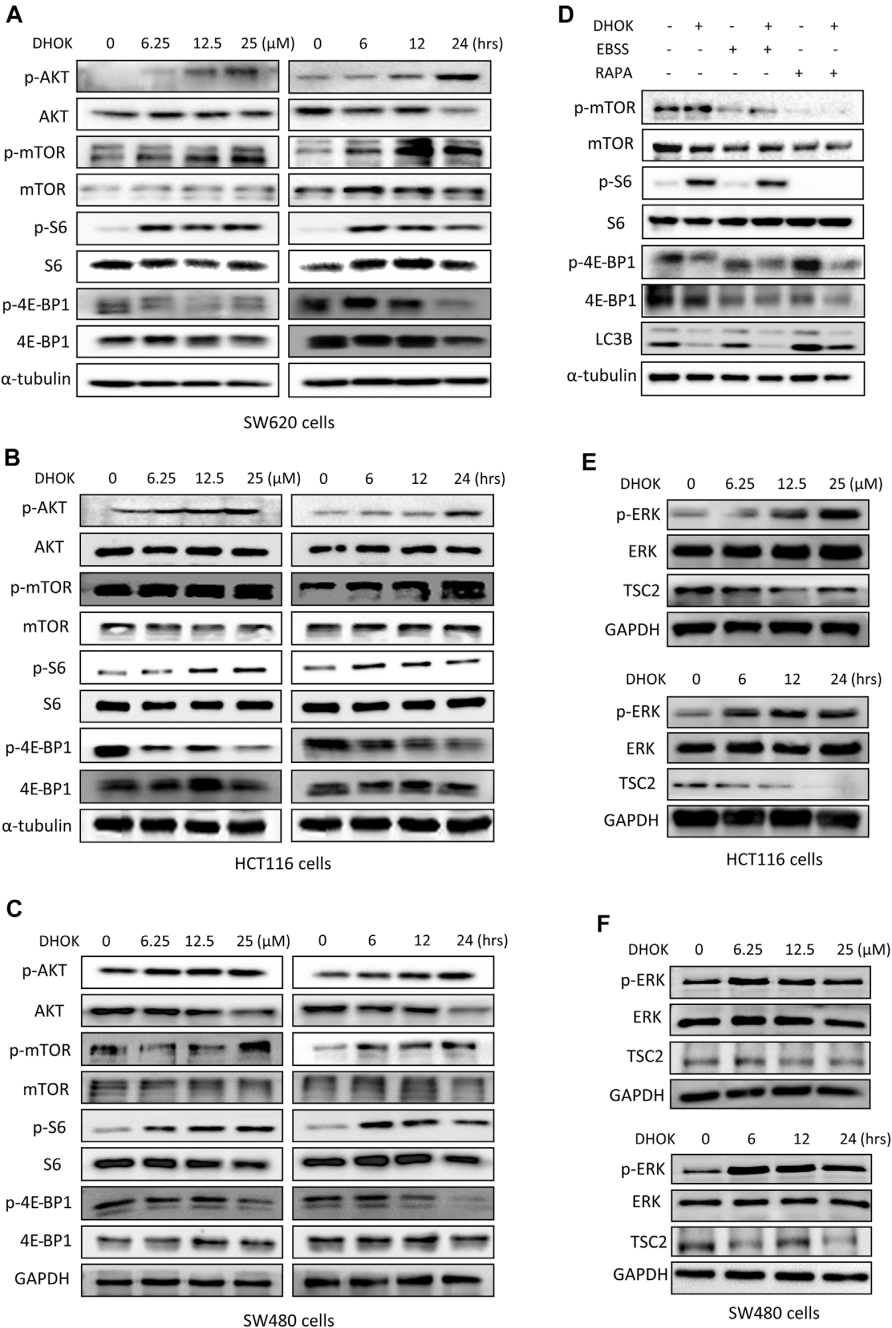
DHOK inhibits autophagy through activation of mTOR signaling pathway. **(A)** SW620 cells were treated with different dosage of DHOK (6.25, 12.5, 25 μM) for different time (6, 12, 24 h). Then cells were lysed for western blotting to detect the phosphorylation levels of AKT, mTOR, S6, and 4E-BP1. α-tubulin or GAPDH served as loading control. **(B,C)**, as in **(A)**, HCT116 and SW480 cells were treated with DHOK and cells were harvested for western blotting analysis of the above phosphorylated proteins. **(D)** SW620 cells were pretreated with EBSS starvation or rapamycin (100 nM) for 2 h and then treated with DHOK (12.5 μM, 24 h). And then cells were harvested for western blotting analysis of the phosphorylated mTOR, S6, 4EBP1, and LC3 levels. α-tubulin was used as loading control. **(E,F)** HCT116 and SW480 cells were treated with different dosage of DHOK (6.25, 12.5, 25 μM) for different time (6, 12, 24 h). And then cells were lysed for western blotting to examine the phosphorylated-ERK, ERK, and TSC2 protein levels. GAPDH was used as loading control.

To reveal the molecular mechanism of autophagy inhibition by DHOK, we focused on the changes of mTOR signaling pathway, a classical pathway regulating autophagy. Here, SW620 cells were chosen to be treated with different dosages of DHOK for different time and western blot results showed the increase of an array of phosphorylated proteins (Figure 4A), including AKT, mTOR, S6 ribosomal protein and 4E-BP1, which belongs to AKT-mTOR signaling pathway. It suggested that DHOK treatment activates the AKT-mTOR signaling pathway in a dose- and time-dependent manner, which may be responsible for DHOK-caused autophagy inhibition. Consistently, the increase of these phosophorylated proteins were also detected in other human CRC cells, including SW480 and HCT116 cells (Figures 4B,C). Moreover, we treated cells with mTOR inhibitors to determine the active effect of DHOK on mTOR pathway. As shown in Figure 4D, either EBSS starvation or rapamycin treatment decreased the phosphorylation levels of mTOR, S6, and 4E-BP1, leading to the increase of autophagy level. But addition of DHOK reversed the inhibition of mTOR signaling pathway to some extent and also attenuated the increase of autophagy level by starvation or rapamycin. It demonstrated that DHOK-caused autophagy inhibition can be attributed to mTOR activation.

In addition, we also investigated the upstream of mTOR and revealed the role of another molecule ERK in the activation of mTOR pathway by DHOK. Similar to AKT changes, in both HCT116 and SW480 cells, the phosphorylation level of ERK was increased with DHOK treatment in a time- and dose-dependent manner (Figures 4E,F). Meanwhile, its downstream molecule TSC2 was decreased accordingly, leading to the activation of mTOR signaling. But in SW620 cells, there was no significant increase in phosphorylation level of ERK levels after 12 h or 24 h treatment. Because the phosphorylation of ERK is a relatively fast process (Waas et al., 2003) thus the detection time point should be earlier. Our results showed that the phosphorylation level of ERK was greatly increased within a short time after DHOK treatment, while TSC2 level was also significantly decreased after 6 h treatment (Supplementary Figure S1B). In addition, we also examined the effect of DHOK on another two MAPK family members, p38 and JNK. The results showed that DHOK also increased the phosphorylation levels of p38, and JNK (Supplementary Figure S1C).

It seemed that DHOK regulates mTOR signaling through multiple pathways.

### Autophagy Protects Cancer Cells From the Cytotoxicity of DHOK

**FIGURE 5:**
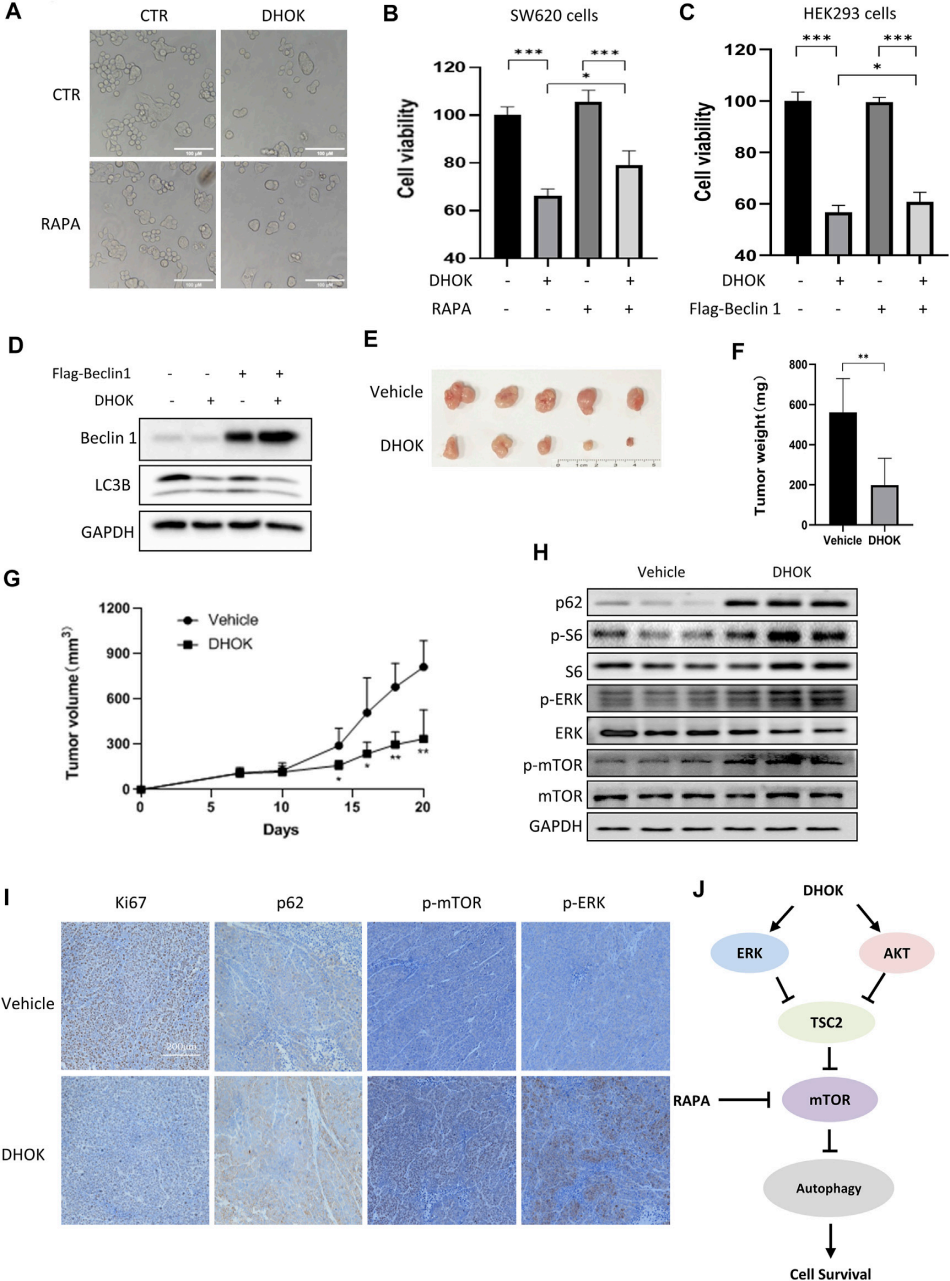
DHOK suppresses human CRC cell growth and tumorigenesis through autophagy inhibition. **(A)** Cell morphology of SW620 cells under DHOK treatment (12.5 μM, 24 h) in the presence or absence of rapamycin (100 nM). Scale bar 100 μm. **(B)** as in **(A)**, cell viability of SW620 cells under DHOK and rapamycin treatment. *p < 0.05 ***p < 0.001 **(C)** HEK293 cells were first transfected with Flag-Beclin 1 and then treated with DHOK (12.5 μM, 24 h). Cell viability was measured using MTT assay ***p < 0.001 **(D)** as in **(C)**, cells were collected and lysed for western blotting analysis of Beclin 1 and LC3. **(E, F)** Xenograft tumors were established by subcutaneously injecting SW620 cells and treated with or without DHOK (40 mg/kg). After 20 days inoculation, all mice were sacrificed and tumor mass was removed, photographed and weighed. **p < 0.01 **(G)** Xenograft tumor volumes were measured and tumor growth curves were plotted. *p < 0.05 **p < 0.01 **(H)** Western blotting analysis of p62, phosphorylated S6, ERK, and mTOR protein levels in xenograft tumor tissues from the Vehicle and DHOK treatment groups. GAPDH was used as loading control. **(I)** Xenograft tumor tissues were collected and prepared for immunohistochemistry analysis of Ki67, p62, phosphorylated ERK and mTOR levels (scale bar: 200 µm). **(J)** Schematic diagram of the anti-cancer mechanism of DHOK.

To elucidate the role of autophagy in DHOK-caused cell growth inhibition, we first treated cells with autophagy inducer rapamycin to activate autophagy. Cell morphology results showed less cell death under DHOK treatment in the presence of rapamycin (Figure 5A). The similar results were also shown in MTT assay, in which autophagy activation protected cell viability under DHOK treatment, indicating that autophagy serves as cell survival (Figure 5B). In addition, we also performed genetic induction of autophagy to confirm the survival role of autophagy through autophagy key gene Beclin 1 overexpression. As shown in Figures 5C,D, Beclin 1 was transfected to induce autophagy and overexpression of Beclin 1 significantly attenuated the cytotoxicity of DHOK.

Finally, xenograft model mice were intraperitoneally treated with DHOK to evaluate the antitumor effect of DHOK *in vivo*. During the experiment, all tumor-bearing mice were alive and no mice died. Also there was no significant difference observed in body weight between the vehicle and DHOK groups in the first of 14 days. With time, the body weight was slightly decreased in the vehicle group, while the body weight was significantly increased in the DHOK group (Supplementary Figure S1D). After 20 days inoculation, all mice were sacrificed (Supplementary Figure S1E) and tumor mass was removed, photographed and weighed (Figures 5E,F). As shown in Figure 5G, treatment with DHOK inhibited tumor xenograft growth by 64.6% when compared with the vehicle. This result indicated that DHOK also exerts a tumor-suppressive effect *in vivo*. In addition, we also examined autophagy-related protein expression in tumor tissue by western blotting and immunohistochemistry. The results showed that autophagy protein p62 level was upregulated and Ki67 level was downregulated in the DHOK group (Figures 5H,I), indicating the decrease of autophagy. Meanwhile, the phosphorylated S6 level, the phosphorylated ERK level and the phosphorylated mTOR level were increased after DHOK treatment (Figures 5H,I), indicating the activation of mTOR signaling, which was consistent with the above *in vitro* results.

## Discussion

*E. longifolia Jack*, also named tongkat ali, is one of Malaysia’s three national treasures. Many compounds extracted from *E. longifolia* have been found the activity of anti-cancer. In general, the compounds in *E. longifolia* exert anti-cancer effects mainly through apoptosis and cell cycle arrest. For example, F16 (an active component of Eurycoma longifolia Jack) induced apoptosis in MCF-7 cells (Tee and Azimahtol, 2005); eurycomanone induced apoptosis and G2/M arrest in HepG2 (Zakaria et al., 2009); TAF273 (a fraction of *E. longifolia* root methanolic extract) induced apoptosis and cell cycle arrest in K-562 cells (Al-Salahi et al., 2014); a standardized quassinoid mixture (SQ40) from *E. longifolia* inhibited LNCaP cell growth through G0/G1 phase arrest (Tong et al., 2015). DHOK is a quassinoid isolated from *E. longifolia*. Although DHOK has been reported to have anti-cancer effects *in vitro*, the mechanism remains unclear. Here, we first found that DHOK can inhibit CRC cell proliferation but does not induce apoptosis (Figure 1). To elucidate the anti-cancer mechanism of DHOK, we did RNA-seq and then performed GO analysis and KEGG analysis. The results indicated DHOK may influence autophagy-related signaling pathways (Figure 2). After that, we determined autophagic flux and confirmed the inhibitory effect of DHOK on atuophagy and lysosomal function (Figure 3).

Autophagy is an intracellular nutrient scavenging pathway, in order to sustain survival in starvation, cells eat parts of themselves and presumably recycle the breakdown products (Guo and White, 2016). Autophagy is a complicated regulatory process, which is regulated by many upstream regulating signaling pathways, such like PI3K-AKT-mTOR, LKB1-AMPK-mTOR, P53, Beclin 1, and Bcl-2 pathway (Chen et al., 2010). Among these pathways, mTOR serves as the main regulator of autophagy (Chen et al., 2010), meanwhile, it is key regulators of translation and ribosome biogenesis, and it contributes to autophagy induction in response to starvation (Jung et al., 2010). Therefore, we examined the effect of DHOK on the mTOR pathway. The results showed an array of phosphorylated proteins increased in CRC cells, which belong to AKT-mTOR signaling pathway, including AKT, mTOR, S6 ribosomal protein, and 4E-BP1 (Figures 4A–C). DHOK can reverse the inhibition of mTOR signaling pathway by starvation or rapamycin and can attenuate the increase of autophagy level, which demonstrated that DHOK-caused autophagy inhibition can be attributed to mTOR activation (Figure 4D). We also investigated the upstreams of mTOR and found that the activation of MAPK pathway may lead to the enhancement of mTOR signaling through inhibiting TSC2 (Figures 4E,F, Supplementary Figures S1B,C). *In vivo* experiment results also support this conclusion (Figures 5H,I).

The function of autophagy in physical process is complexand defects in autophagy affects mammalian survival. For example, Atg5 is a key autophagy gene and essential for autophagosome formation, and Atg5 knockout mice died within 1 day of delivery (Kuma et al., 2004). Thus, autophagy gives hungry newborn mice energy to live longer. When it comes to cancer, the function of autophagy is still in an undetermined debate. Autophagy in cancer is either tumor suppression or tumor promotion. The tumor suppressive function of autophagy was firstly validated through genetic approaches of Beclin 1 (Chen et al., 2010). Yue et al. discovered that Beclin 1 knockout mice died early in embryogenesis while a high incidence of spontaneous tumors occurred in Beclin 1^+/−^ mutant mice, supporting the tumor suppression activity of autophagy (Yue et al., 2003). Bax-interacting factor-1 (Bif-1) protein is another positive regulator of autophagy and loss of Bif-1 promotes colon adenocarcinomas (Coppola et al., 2008). All of these data support the role of autophagy in tumor suppression.

There is growing evidence that autophagy supports tumor survival (Chen et al., 2010). Allelic loss of Beclin 1 results in the sensitization of mammary epithelial cells to metabolic stress and lumen formation in mammary acini is also accelerated (Karantza-Wadsworth et al., 2007). In Bax/Bak-deficient cells depending on growth factor, autophagy protects the cell death under the deprivation of growth factor (Lum et al., 2005). Through recycling the damaged proteins and organelles, autophagy serves as a back-up energy reserve to sustain the survival of cancer cells against different cancer therapeutics (Chen et al., 2010). Therefore, autophagy may play a tumor-promoting role by alleviating and limiting metabolic stress to protect the integrity of the genome and develop resistance to anti-cancer therapy.

Beclin 1 participates in the initial stage of autophagy and is essential for the autophagosome formation (Cao and Klionsky, 2007; Chen et al., 2010). In our experiment, autophagy was activated by transfection of Flag-Beclin 1 (Figure 5C). We found that DHOK has an inhibitory effect on autophagy, and autophagy protect tumor cells (Figures 5A,B,D). As we know, many traditional Chinese medicine extracts or compounds have also been shown to induce autophagy, such as curcumin (Shinojima et al., 2007), dihydroartemisinin (Hu et al., 2014), and artesunate (Zhou et al., 2020). And only a few compounds have an inhibitory effect, such as 3-Methyladenine, bafilomycin, blocking the fusion of and CQ. These autophagy inhibitors have their own disadvantages, for example, 3 MA requires a higher working concentration and is more toxic to cells; CQ can induce retinal damage (Udristioiu and Nica-Badea, 2019); bafilomycin can induce apoptosis (Yuan et al., 2015). Unlike these inhibitors, DHOK seems to have fewer side effects. As shown, DHOK did not induce cell apoptosis (Supplementary Figure S1A), and *in vivo* experiments demonstrated the safety of DHOK treatment in tumor-bearing nude mice (Supplementary Figure S1D). Therefore, DHOK has the great potential to be developed as a novel autophagy inhibitor.

It has been revealed in many preclinical studies that various cancer therapies are also able to induce autophagy, which serves as cytoprotective rather than cytotoxic (Onorati et al., 2018). For example, a proteasome inhibitor bortezomib has exerted broad anti-tumor activities in many malignancies while it also induces protective autophagy in cancer cells (Min et al., 2014). Through reserving the energy, autophagy helps cancer cells to build resistance to metabolic stress caused by different cancer therapeutics (Chen et al., 2010). Thus, autophagy inhibitors have been widely used in the treatment of cancer. As an autophagy inhibitor, DHOK has the possibility of clinical application. Of course, this requires more experiments and more experimental data to support. For example, detailed toxicology studies are also needed to confirm safety. Meanwhile, pharmacokinetics study of DHOK is also warranted.

In summary, we have elucidated that autophagy inhibition is an inhibitory mechanism of DHOK against CRC cell proliferation (Figure 5J). The autophagy is controlled by the mTOR pathway, suggesting that agonists of the mTOR pathway, including DHOK, could be a potential therapeutic strategy for CRC. Considering that many anti-cancer drugs activate protective autophagy, combination of DHOK and other drugs is also full of potential, and we will do subsequent experiments.

## Data Availability

The datasets presented in this study can be found in online repositories. The names of the repository/repositories and accession number(s) can be found below: Gene Expression Omnibus–GSE189697.
