# Supplementary figures and images for: DHOK Exerts Anti-Cancer Effect Through Autophagy Inhibition in Colorectal Cancer
Source: Front Cell Dev Biol. 2021 Dec 17;9:760022. doi: 10.3389/fcell.2021.760022 (PMC8719673; doi:10.3389/fcell.2021.760022)

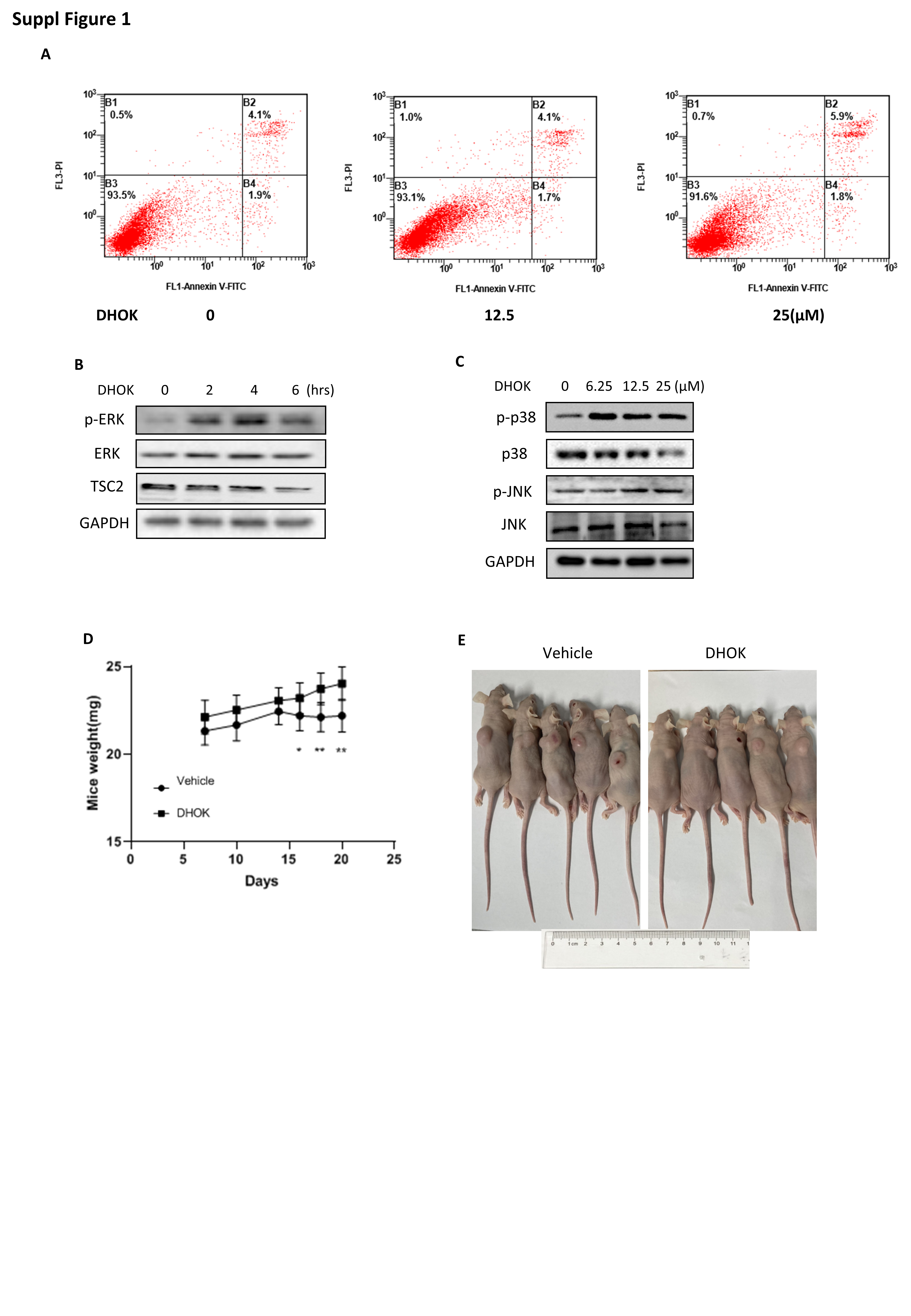

Supplement: Supplementary file 1 [file Image1.TIF]
